# A comprehensive graph neural network method for predicting triplet motifs in disease–drug–gene interactions

**DOI:** 10.1093/bioinformatics/btaf023

**Published:** 2025-01-20

**Authors:** Chuanze Kang, Zonghuan Liu, Han Zhang

**Affiliations:** College of Artificial Intelligence, Nankai University, Tianjin 300350, China; College of Artificial Intelligence, Nankai University, Tianjin 300350, China; College of Artificial Intelligence, Nankai University, Tianjin 300350, China

## Abstract

**Motivation:**

The drug–disease, gene–disease, and drug–gene relationships, as high-frequency edge types, describe complex biological processes within the biomedical knowledge graph. The structural patterns formed by these three edges are the graph motifs of (disease, drug, gene) triplets. Among them, the triangle is a steady and important motif structure in the network, and other various motifs different from the triangle also indicate rich semantic relationships. However, existing methods only focus on the triangle representation learning for classification, and fail to further discriminate various motifs of triplets. A comprehensive method is needed to predict the various motifs within triplets, which will uncover new pharmacological mechanisms and improve our understanding of disease–gene–drug interactions. Identifying complex motif structures within triplets can also help us to study the structural properties of triangles.

**Results:**

We consider the seven typical motifs within the triplets and propose a novel graph contrastive learning-based method for triplet motif prediction (TriMoGCL). TriMoGCL utilizes a graph convolutional encoder to extract node features from the global network topology. Next, node pooling and edge pooling extract context information as the triplet features from global and local views. To avoid the redundant context information and motif imbalance problem caused by dense edges, we use node and class-prototype contrastive learning to denoise triplet features and enhance discrimination between motifs. The experiments on two different-scale knowledge graphs demonstrate the effectiveness and reliability of TriMoGCL in identifying various motif types. In addition, our model reveals new pharmacological mechanisms, providing a comprehensive analysis of triplet motifs.

**Availability and implementation:**

Codes and datasets are available at https://github.com/zhanglabNKU/TriMoGCL and https://doi.org/10.5281/zenodo.14633572.

## 1 Introduction

In the biomedical knowledge graph (BKG), the relationships between disease, gene (or protein), and drug entities, as the high-frequency edge types, help us to understand complex biological processes (Li *et al.* 2022a,b, Huang *et al.* 2024a). The pairwise relationship among the three implies rich pharmacological mechanisms. Specifically, estradiol treats the breast cancer (BC) by stabilizing p53 protein in MCF-7 cell line (Okumura *et al.* 2002). Trastuzumab, a recombinant antibody targeting HER2 gene, was the first biological drug approved for the treatment of HER2-positive BC (Maximiano *et al.* 2016). Imatinib treats chronic myeloid leukemia (CML) by inhibiting the BCR-ABL1 fusion protein that is a deregulated tyrosine kinase resulting from the Philadelphia chromosome (Quintás-Cardama and Cortes 2009).

**Figure 1.**
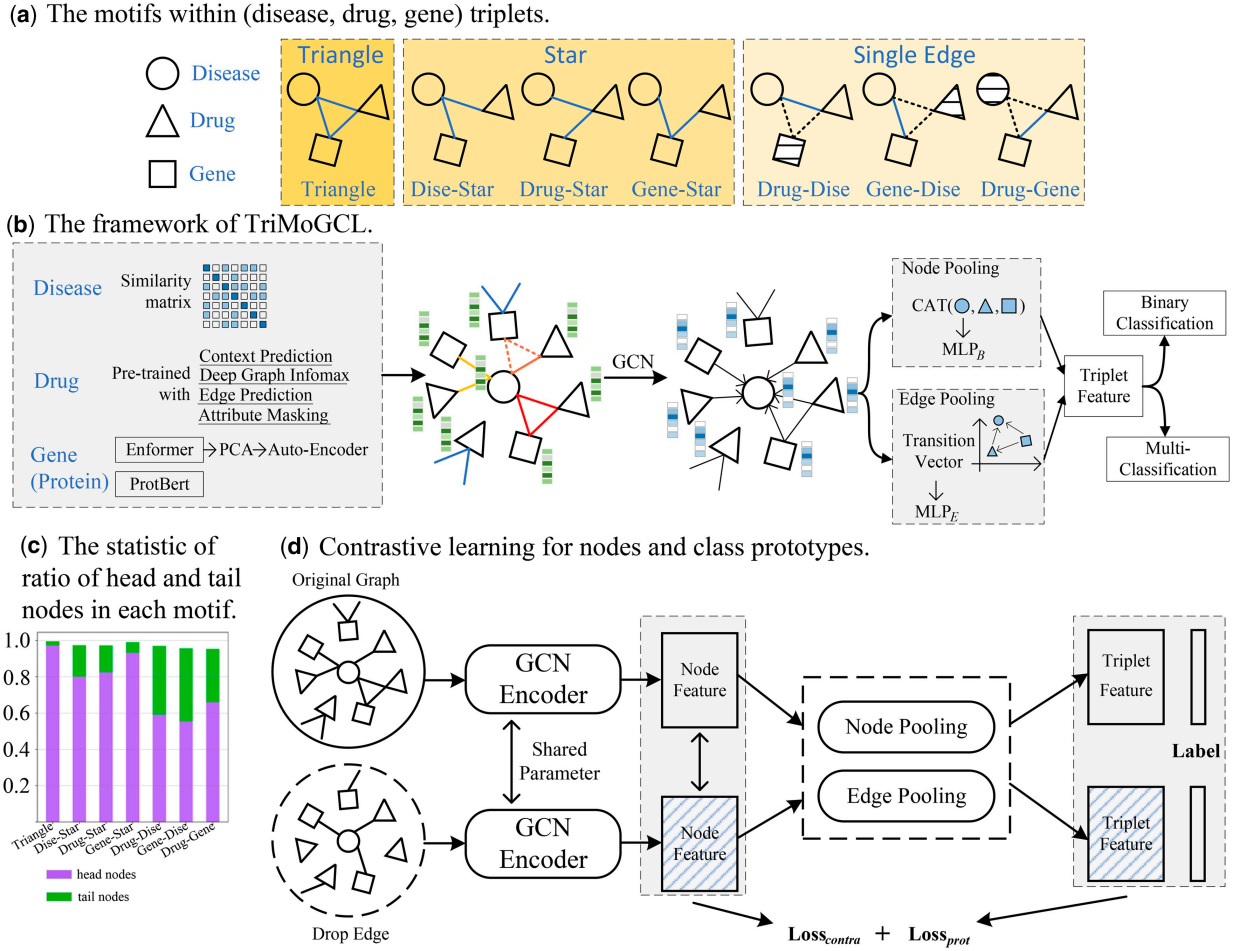
(a) The seven motif types within the (disease, drug, gene) triplets. (b) The overall framework of TriMoGCL. (c) The statistic of the ratio of head and tail nodes in each motif. High ratio of tail nodes means that the triplets have sparse context information. (d) The architecture of contrastive learning for nodes and class prototypes.

However, when drug A acts on gene B and B regulates disease C, we intuitively would assume that the drug would treat disease C. In fact, according to a statistical analysis, only 6% and 3% of the triplets conform to the assumed rule above when the current pairwise relationship exists in DRKG and MS dataset (Ioannidis *et al.* 2020, Ruiz *et al.* 2021). That is, the triangular relationship is a relatively rare and strong structural pattern in the BKG. It cannot be identified simply by inference using composition rules, so advanced classification methods are required to recognize complex structural motifs within disease–drug–gene triplets. According to the structural pattern (Besta *et al.* 2022) within the [disease (dise), drug, gene] triplets, the triplet motifs are classified into seven types: triangle, dise–star, drug–star, gene–star, drug–dise, gene–dise, and drug–gene, as shown in Fig. 1. The triangle motif represents the full connectivity within the triplet. The star motif represents a triplet that contains two rays centered on one node. Single-Edge represents a triplet containing only one edge. The nonexisting relationships in a triplet may represent the biological blockages that are also crucial in medical research. For instance, the triplet {BC, Lasofoxifene, p53} is the dise–star (Damodaran *et al.* 2023). The nonexisting relationship between lasofoxifene and p53 indicates that lasofoxifene did not act on the p53. The triplet {CML, Estradiol, BCR-ABL1} is the gene–dise single-edge. The nonexisting relationships represent that estradiol did not treat CML and act on BCR-ABL1 protein. Thus, we consider constructing the complex distribution of triplet motifs with triangle and other six types. Moreover, the prediction of seven motifs is not only conducive to mining the property of triangular relationships, but also convenient for discovering new triangular relationships (i.e. pharmacological mechanism) by distinguishing the semantics between different motifs.

The current research has already focused on the triangle motif. Sheng *et al.* (2023) and Huang *et al.* (2024b) used graph contrastive learning to extract topological features of nodes on lncRNA–miRNA–disease heterogeneous graphs for prediction of associations among lncRNAs, miRNAs, and diseases. Though they investigated three types of associations between the three nodes, they did not identify the motifs of the triplets. Liu *et al.* (2023) studied drug–microbe–disease (DMD) relationship prediction. They merge the drug-microbe and microbe–disease associations into the data schema: [drug, microbe, disease], which makes the DMD triplets cover the triangle and star motifs. Due to the sparse number of collected triplets, they proposed a multi-view hypergraph contrastive learning method to extract robust node features. Single-edge motifs are selected as the negative samples corresponding to DMD triplets. Due to the scarcity of DMD relations, they ignored complex motif prediction within DMD triplets as well.

Triplet relationships and triangular representation learning have been widely studied. In the field of graph machine learning, Chavan and Potika (2020) studied the co-occurrence of three nodes and considered triangles as a special interaction of three nodes, where three triangle embedding methods are proposed for the binary classification of triangle motif. In the recommendation system, Jiang *et al.* (2022) considered triangles as the basic unit of user interest in item-item graphs. They considered Intra-triangle homophily and Inter-triangle heterophily to build a Triangle Graph Interest Network (TGIN) for learning item features. So, all the above methods focus on the prediction of a single motif and fail to further discriminate various motifs of triplets.

SEAM algorithm has been proposed for graph motif prediction with graph neural network (Besta *et al.* 2022). Given a set of nodes in the network, SEAM can predict whether their edges form a motif of interest to the user (e.g. 5-clique). Although SEAM considers diverse motifs from dense to sparse, it does not focus on identifying which motif a triplet belongs to. Consequently, there is no suitable deep-learning algorithm for the multi-classification of the seven motifs within triplets.

In this paper, we propose a triplet motif prediction method with a graph neural network to identify the motifs within the triplets composed of disease, drug, and gene (or protein). Firstly, a graph convolutional encoder is used to extract node features based on the topology of the global network, where the types of all nodes are viewed as the same in the network. Secondly, we use node pooling and edge pooling to construct features for the triplet from global and local perspectives. Specifically, node pooling fuses the global node features into the context information of triplets, and edge pooling considers local interaction features within triplets as another semantic information of triplets. Furthermore, due to the dense edges in the network, the triplets of the triangle and star motifs suffer from the redundancy of context information and motif imbalance problems. Thus, node and class-prototype contrastive learning are used to reduce redundancy, enabling the model to distinguish semantics between motifs and represent triplets containing different neighbor information. For the experiments, we select two knowledge graphs with different scales and only extract the edges between disease, drug, gene (or protein) nodes from graphs. Binary-classification and multi-classification tasks are performed on the seven motifs. Compared to other methods, our model achieves the best performance in all tasks. In the result analysis, we further explore the classification paradigm of motifs and the different roles of features from node pooling and edge pooling. Specifically, the triplets of triangle motif typically mean high neighbor similarity, and it is commendable that TriMoGCL can still achieve the prediction of 0-similarity triplets through GNN and disease similarity matrix. In the case study, our model discovers new pharmacological mechanisms by triangle-based binary classification.

## 2 Materials and methods

### 2.1 Dataset

*Drug Repurposing Knowledge Graph (DRKG)* is a large comprehensive biological knowledge graph that involves genes, drugs, diseases, biological processes, side effects, and symptoms. It includes 97 238 entities belonging to 13 entity types; and 5 874 261 triplets belonging to 107 edge types (Ioannidis *et al.* 2020).

*Multi-scale interactome (MS) network* is a small heterogeneous knowledge graph containing drug, disease, protein, and gene ontology biological function nodes. The MS consists of 29 959 nodes belonging to 4 node types and 478 728 edges belonging to 4 edge types (Ruiz *et al.* 2021).

We extract edges between disease, drug, and gene (protein) entities from two-scale networks. The statistics of node and edge information are provided in Table 1. The construction of initial features for node representation learning is as follows. For disease entities, the similarity matrix between diseases as initial feature is computed based on the directed acyclic graph of diseases in MESH (Li *et al.* 2020). For drug entities, the molecular graph is constructed based on SMILES strings, and the features of drug atoms are computed using the pre-trained GIN model on the DGL-LifeSci platform (Chen *et al.* 2021, Yu *et al.* 2023). Afterward, the molecular features are obtained by average pooling of the atomic features. We concatenate the features derived from four pre-trained ways as the initial features of the drugs. For the gene nodes, we extract the corresponding sequences in chromatin from the NCBI website based on their Gene ID. The pre-trained model Enformer predicts 5313 genomic tracks for the human genome and 1643 tracks for the mouse genome, each length of 896 corresponding to 114 688 bp aggregated into 128-bp bins (Avsec *et al.* 2021). Then, PCA is used to reduce the number of genomic tracks from 5313(1643) to 100 and each gene obtains a feature matrix with dimensions of 896×100. We convert the matrix into 1D vectors using a convolutional neural network-based auto-encoder as the initial features of the genes. The pseudo-code of constructing initial gene features is shown in the Supplementary Data (SD).

**Table 1.** The statistics of DRKG and MS datasets in one triplet sampling.^a^

Dataset	Dise	Drug	Gene	Edges	Triangle	Dise–star	Drug–star	Gene–star	Drug–dise	Gene–dise	Drug–gene	Des.	CC
DRKG	2157	2908	9809	199 745	13 335	8856	15 180	20 975	3758	36 535	29 425	0.4%	0.26
MS	694	1272	4519	31 733	450	1658	2217	1669	296	15 983	2593	0.3%	0.067

a Des.: Density of network, CC: Clustering Coefficient.

### 2.2 Notation and problem formulation

Let the biomedical network be G=(V,E), where *V* is the node set and includes gene, drug, and disease nodes. *E* is the edge set that includes drug–disease, gene–disease, and drug–gene edges. *X* is the initial node feature. Since the length of features varies from node type, we pad shorter features with zeros to match the longest dimension. We define a triplet motif as the structural pattern formed by edges between three entities. Seven structural patterns are considered as triplet motifs as shown in Fig. 1: triangle $M_{1}$, dise–star $M_{2}$, drug–star $M_{3}$, gene–star $M_{4}$, drug–dise $M_{5}$, gene–dise $M_{6}$, and drug–gene $M_{7}$. We provide the problem formulation of motif classification as follows:

Given an initial feature *X*, biomedical knowledge graph *G*, a set of triplets $\left\{t|t=\left(\mathrm{dis}e_{i},\mathrm{dru}g_{j},\mathrm{gen}e_{k}\right)\right\}$, a set of motif structure $Ms=\{M_{1},\ldots ,M_{7}\}$,

- **Binary-classification problem** needs a function $p_{t}^{M}=f\left(t|\theta ,\theta_{bc},X,G\right)$ to predict the probability that the motif type of *t* is M (M∈Ms).
- **Multi-classification problem** needs a function $p_{t}=f\left(t|\theta ,\theta_{mc},X,G\right)$ to predict the probability of each motif type to which *t* belongs.

θ is the parameters of the feature encoder. $\theta_{bc}$ and $\theta_{mc}$ are the parameters of classification hyperplanes respectively.

### 2.3 Overview

To solve the motif prediction problem for disease–drug–gene triplets, we propose a graph neural network method with contrastive learning. Firstly, we regard the types of all nodes as the same and exploit a graph convolutional encoder to extract node features based on the topology of the global network. Secondly, node pooling extracts the context information of triplets with node features from the global view. Then, a new latent space is constructed by transition vectors of edges to represent triplets with semantic information from the local view. Furthermore, due to the dense edges in the network, the triplets of the triangle and star motifs suffer from context information redundancy and motif imbalance problems. Thus, we design the node and class-prototype contrastive learning to denoise triplet features, which enables the model to distinguish semantics between motifs and represent triplets containing sparse neighbor information.

### 2.4 Graph convolutional network

To extract topological information from the global view, we regard the types of all nodes as the same and use graph convolution network (Kipf and Welling 2016) to obtain the node feature:

$$H^{\left(l+1\right)}=\sigma \left(D^{-1/2}AD^{-1/2}H^{\left(l\right)}W_{1}\right),$$

where *A* is the adjacent matrix of graph *G*, $D^{-1/2}$ is the diagonal matrix, *D_ii_* is the degree $\sum_{j}\underset{\mathrm{ij}}{A}$, $D^{-1/2},\;\sigma$ is the activation function, and $H^{\left(0\right)}=X$. $W_{1}$ and $W_{2}$ are the trainable parameter matrices and are applied on node features for linear transformation. We simplify the above equation as follows:

(1) $$H=GCN_{2}\left(GCN_{1}\left(X,G\right),G\right),$$

where $GCN_{i}$ denotes the *i*th layer graph convolution.

### 2.5 Node pooling

$GCN_{2}$ aggregates the 2-hop neighbor information in the knowledge graph as a feature of entities. Then a pooling operation is used to fuse the neighbor information of the node into the context information of triplets. Node pooling concatenates the features of three nodes:

(2) $${\tilde{Z}}_{t}^{B}=\mathrm{Cat}\left(H\left[\mathrm{dis}e_{i}\right],H\left[\mathrm{dru}g_{j}\right],H\left[\mathrm{gen}e_{k}\right]\right),$$

and extract the triplet representation by a multi-layer fully connected network

(3) $$Z_{t}^{B}=MLP_{B}\left({\tilde{Z}}_{t}^{B}\right).$$

H[index] indicates *index*-th row of *H* matrix. All triplets pass through one feature filter so that the latent embedding space obtains knowledge shared between classes.

### 2.6 Edge pooling

However, node pooling extracts context information from the global view, and does not consider the local interaction features between nodes. Triangle lasso computes the distances of connected nodes in the triangle motif as a regularization loss (Zhao *et al.* 2019). Inspired by the Triangle Lasso, we consider interaction relations between nodes to compute the local context information of triplets. We define the transition vector between two nodes as:

(4) $$e_{di\to dr}=H\left[\mathrm{dis}e_{i}\right]-H\left[\mathrm{dru}g_{j}\right],$$

 

(5) $$e_{di\to ge}=H\left[\mathrm{dis}e_{i}\right]-H\left[\mathrm{gen}e_{k}\right],$$

 

(6) $$e_{dr\to ge}=H\left[\mathrm{dru}g_{j}\right]-H\left[\mathrm{gen}e_{k}\right].$$

Faced with different triplets, three transition vectors can effectively provide distinguishable semantic information different from that obtained by node features. Then, we project all transition vectors to a new latent space to represent the triplet in another view. Three transition vectors are concatenated after nonlinear transformation:

(7) $${\tilde{Z}}_{t}^{E}=\mathrm{Cat}\left(\sigma (e_{di\to dr}W_{E}),\sigma (e_{di\to ge}W_{E}),\sigma (e_{dr\to ge}W_{E})\right),$$

where $W_{E}$ is the trainable parameter matrix.

All triplets pass through a feature filter as well:

(8) $$Z_{t}^{E}=MLP_{E}\left({\tilde{Z}}_{t}^{E}\right).$$

Through edge pooling, different semantic features between triplets are obtained from the local view. Then two triplet features are effectively constructed considering only the node features and interaction relations. The final representation of triplet *t* is:

(9) $$Z_{t}=\mathrm{Cat}\left(Z_{t}^{B},Z_{t}^{E}\right).$$

### 2.7 Contrastive learning

Due to the complex distribution of motifs, there are two important challenges when predicting triplet motifs. Firstly, triangle and star motifs frequently appear in dense areas of the network, and dense edges cause redundancy in context information. The triangle motif is a closed graph structure, but the structure of the star motif is open. Redundant edges cause open triplets to obtain similar information of closed triangle through graph convolution, which makes the model confuse the motifs of these triples. Secondly, for triplets with sparse context information, the number of triplets from triangle and star motifs is much smaller than that of triplets from single-edge motifs, which leads to a motif imbalance problem. Due to the lack of sufficient data samples, the model tends to believe that triplets composed of nodes with sparse neighbors would not form triangle and star motifs. Therefore, graph contrastive learning (You *et al.* 2020) is used to construct another view by randomly removing some edges, and make the node features of the two views close. By reducing redundancy, the model can refine the context information of triplets of triangle and star motifs, and simultaneously enhance its ability to learn features for triplets with sparse context information.

$$\begin{array}{c} A^{\prime }=A⊙M,\\ H^{\prime }=\mathrm{GCN}\left(A^{\prime },X\right). \end{array}$$

where $A^{\prime }$ is the adjacent matrix of another graph view obtained by randomly dropping edges of the original graph *G*. *M* is the mask of edges to be dropped. GCN is the graph neural network with the shared parameter matrices $W_{1}$ and $W_{2}$.

(10) $$l\left(H_{i},H_{i}^{\prime }\right)=-\log\frac{\;\text{exp}\;\left(f\left(H_{i},H_{i}^{\prime }\right)/\tau_{1}\right)}{\;\text{exp}\;\left(f\left(H_{i},H_{i}^{\prime }\right)/\tau_{1}\right)+\sum exp(f(H_{i},H_{-}^{\prime })/\tau_{1}}$$

where $H_{i}$ and $H_{i}^{\prime }$ are node features from the original and augmented views, *f* denotes the cosine similarity, $H_{-}^{\prime }$ denotes the negative samples, which are the node features different from *i*, τ is the temperature hyperparameter.

(11) $$\mathrm{los}s_{\mathrm{contra}}=\sum l\left(H_{i},H_{i}^{\prime }\right)$$

For the multi-classification problem, we address motif confusion arising from the redundancy of context information by applying class-prototype contrastive loss (Xu *et al.* 2021). We compute the center of each motif $Z_{m}=\frac{1}{\left|T_{m}\right|}\sum_{t\in T_{m}}Z_{t}$, and prototype contrastive loss makes different classes across views distant from each other and the same class close:

(12) $$l\left(Z_{m},Z_{m}^{\prime }\right)=-\log\frac{\;\text{exp}\;\left(f\left(Z_{m},Z_{m}^{\prime }\right)/\tau_{2}\right)}{\;\text{exp}\;\left(f\left(Z_{m},Z_{m}^{\prime }\right)/\tau_{2}\right)+\sum exp(f(Z_{m},Z_{-}^{\prime })/\tau_{2}}$$

 

(13) $$\mathrm{los}s_{\mathrm{prot}}=\sum l\left(Z_{m},Z_{m}^{\prime }\right)$$

where $T_{m}$ is the triplet set of motif *m*, $Z_{m}$ and $Z_{m}^{\prime }$ are the center of class for the original and augmented views, and $Z_{-}^{\prime }$ represents the center of class different from *m*. The semantic information of the motifs in the latent space is distinguished from a global perspective by prototype contrastive loss. Making the both of node and prototype features similar under dense and sparse graph views enhances the ability of model to discriminate triplets having sparse context information, which alleviates the motif imbalance problem.

The model computes the classification probability of triplet *t*:

$$p_{t}=\mathrm{softmax}\left(Z_{t}W\right).$$

where $W\in R^{F\times 2}$ for the binary-classification problem and $W\in R^{F\times 7}$ for the multi-classification problem.

The final classification loss is:

(14) $$\mathrm{los}s_{\mathrm{pred}}=-\frac{1}{\left(N\right)}\;\sum_{t}\log\sum_{m}p_{t}\left[m\right]*y_{t}\left[m\right],$$

where $y_{t}$ represents the label of the triplet, if *t* belongs to motif *m*, then $y_{t}[m]=1$, otherwise $y_{t}[m]=0$. The final loss is:

(15) $$\mathrm{loss}=\mathrm{los}s_{\mathrm{pred}}+\lambda \mathrm{los}s_{\mathrm{contra}}+\lambda \mathrm{los}s_{\mathrm{prot}}$$

where λ is the hyper-parameter.

## 3 Results

### 3.1 Experiment setup

In this section, we introduce the extraction process of the triplet dataset from the knowledge graph and implementation details.

#### 3.1.1 Dataset

Each edge can form multiple triplets, leading to an explosion of triplet numbers. Therefore, we simplify it into a rule that each edge only appears in one triplet. In this way, however, this rule covers a fraction of the triplets, which introduces biases to the integrity of model evaluation. To this end, we perform 10 triplet samplings for each motif and take the average of the 10 results to evaluate the performance and robustness of the model. Specifically, we first extract all triplets of the triangle, dise–star, drug–star, and gene–star motifs in the network. According to the order of the four motifs mentioned above, the triplets of each motif are iterated in a random order. If all edges within the triplet have not been visited, it will be collected for evaluation. The remaining edges of the network are assigned with disconnected nodes to form the triplets of the single-edge motif. For example, $e=(\mathrm{Dis}e_{i},\mathrm{Dru}g_{j})$ remains in the network, and $\mathrm{Gen}e_{k}$ is sampled from the unconnected neighbors to form a triplet together with *e* to represent the drug–dise motif. For multi-classification (MC), the triplets of each motif are divided into training, validation, and testing sets according to 8:1:1. For binary classification (BC), the negative samples of each motif are composed of other motif’s triplets.

#### 3.1.2 Implementation details

We set the hidden dimension of the model 256, $\tau_{1}=1000$, $\tau_{2}=0.01$, λ=0.1. The parameter analysis for contrastive learning is provided in SD. The learning rates are 5e−4 for DRKG and 1e−4 for MS. The batch size is 5000 for DRKG and 1000 for MS. For the BC task, we select AUC and AUPR as metrics and show the results of each motif. For the MC task, we select mi-AUPR, ma-AUC, ma-F1, and ACC as metrics and show the overall results across motifs. To evaluate the performance of the model for motif classification, we compare the model with some methods including Random Forest (RF), MLP, N2V-MLP (Chavan and Potika 2020), TriSAGE (Chavan and Potika 2020), TriNet (Jiang *et al.* 2022), MCHNN (Liu *et al.* 2023), and SEAM (Besta *et al.* 2022).

### 3.2 Results for binary-classification (BC) of motif

**Figure 2.**
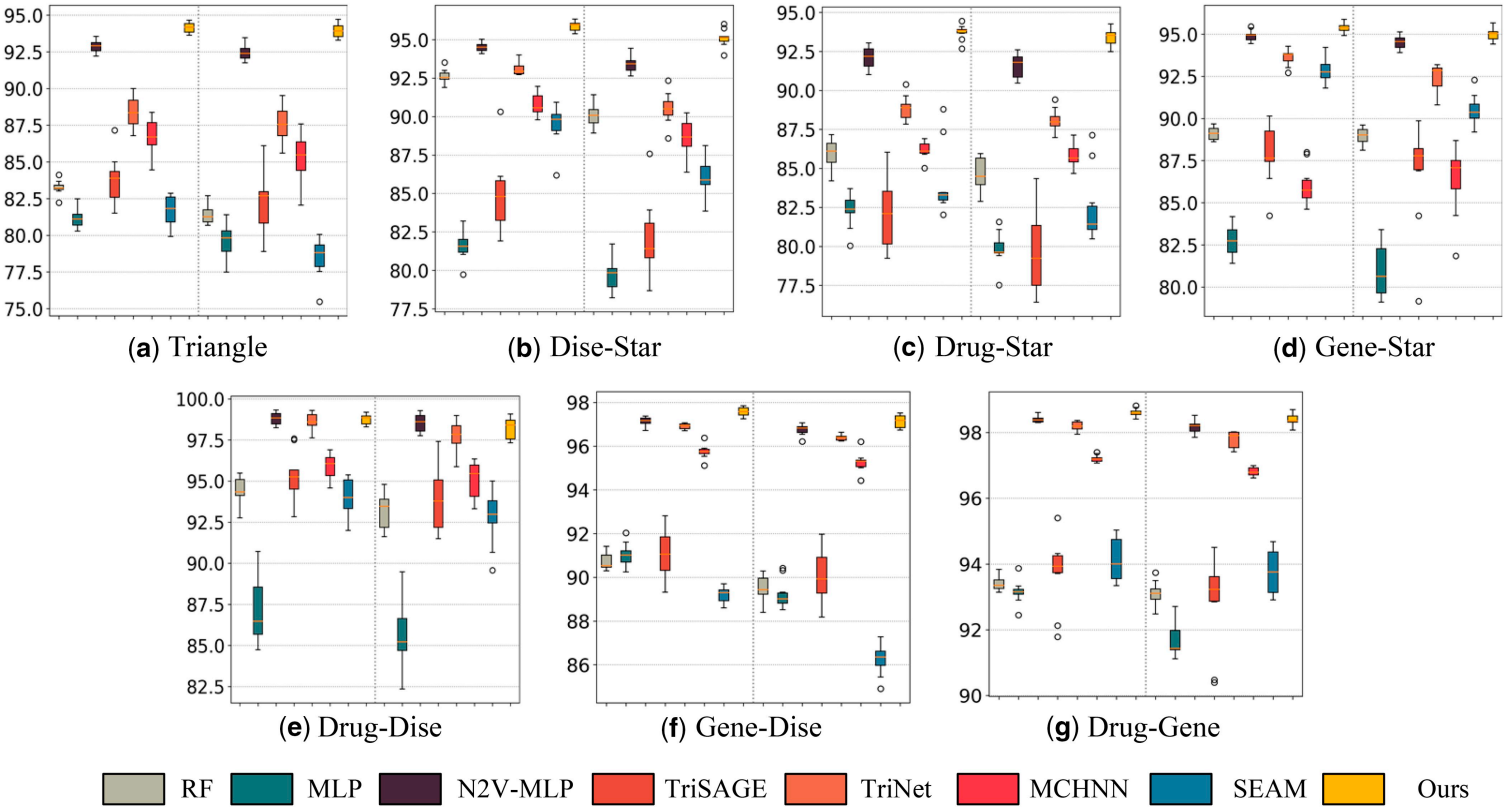
The BC results of TriMoGCL and baselines in DRKG dataset. Subplots (a)–(g) correspond to 7 motifs of triplets. In each subplot, the left plane is the AUC result and the right plane is the AUPR result.

The results of the BC task are shown in Fig. 2 and Supplementary Fig. S1. For the DRKG dataset, TriMoGCL outperforms other methods and has a lower variance. Due to the sparse edges in the MS network, all methods have high variance in results. For drug–dise motifs, genes in triplets may be distant from drugs and diseases, which clearly distinguishes the drug–disease motif from other motifs. Consequently, the results of N2V-MLP, TriNet, and TriMoGCL are good. For triangle, dise–star, drug–star, and gene–star motifs, N2V-MLP, and TriNet have competitive performance. Although N2V-MLP exploits node2vec to obtain node features through long-distance dependence, edge pooling and contrastive learning makes TriMoGCL have better performance. TriNet uses the triplet hypergraph and the multi-head attention to fuse node embedding across motifs. However, learning node embedding from the global topology by GCN helps TriMoGCL to have robust performance.

### 3.3 Results for multi-classification (MC) of motifs

The results of the MC task are shown in Table 2. TriMoGCL achieves the best performance across all metrics in both the MS and DRKG datasets, showcasing its effectiveness and reliability for MC tasks. LP-PP (Hu *et al.* 2020) and NCN-PP (Wang *et al.* 2024) are the state-of-the-art link prediction (LP) methods that independently predict drug–disease, gene–disease, and drug–gene relationships. The likelihoods of triplet motifs are then derived from the probability product (PP) of three relationships. Results show that the LP methods with PP cannot predict complex triplet motifs, so it’s necessary for TriMoGCL to aim at capturing higher-order dependencies and motif-level context. Although N2V-MLP exhibits strong performance across various motifs in the BC task, MLP with initially created features achieves superior performance. TriSAGE is limited to sparse networks, as the noise from dense network edges reduces its effectiveness. Meanwhile, TriNet and MCHNN perform better than SEAM, indicating that the representation ability of hyperedge is superior to that of the sub-graph for MC. Generally, constructing node embeddings from the global network structure proves to be most effective for MC tasks.

**Table 2.** The multi-classification results of model and baselines in MS and DRKG datasets. The bold values indicate the best performance and underlined values represent the second-best performance across all methods for the corresponding metric.

Method	MS				DRKG			
	Micro-AUPR	Macro-AUC	Macro-F1	ACC	Micro-AUPR	Macro-AUC	Macro-F1	ACC
RF	79.64±0.46	66.52±0.75	46.00±1.74	78.08 ± 0.50	65.85±0.43	72.37±0.38	52.26±0.82	63.22±0.46
MLP	80.21±0.76	84.95±0.73	42.44±3.75	75.26±0.70	69.97±0.38	89.55±0.17	54.92±0.76	64.15±0.25
LP-PP	65.67±1.00	79.36±1.21	44.57±1.45	67.14±0.97	68.09±0.23	86.38±0.21	63.77±0.22	69.43±0.21
NCN-PP	57.94±2.31	77.76±1.38	43.05±1.37	63.63±1.73	63.45±1.02	86.63±0.45	61.64±0.76	66.10±0.62
N2V-MLP	60.75±0.83	67.20±1.35	20.61±1.09	64.64±0.52	48.43±0.54	80.08±0.37	40.95±1.00	50.95±0.43
TriSAGE	84.32 ± 0.44	90.11 ± 0.41	49.04±1.22	77.26±0.46	76.87±0.28	92.51±0.09	61.97±0.37	69.78±0.30
TriNet	83.56±0.13	88.06±0.19	50.33 ± 0.51	77.14±0.30	83.89 ± 0.18	95.28 ± 0.06	70.88 ± 0.52	76.36 ± 0.14
MCHNN	83.14±0.34	86.49±0.84	48.87±1.47	77.63±0.46	81.51±0.29	92.96±0.17	70.31±0.73	75.56±0.45
SEAM	74.89±1.19	80.06±1.27	34.06±3.05	71.62±0.91	63.09±1.60	86.00±1.09	44.70±2.84	57.23±1.36
Ours	**92.60** ± **0.14**	**95.39** ± **0.07**	**70.44** ± **0.74**	**85.96** ± **0.25**	**90.00** ± **0.07**	**97.30** ± **0.02**	**78.58** ± **0.18**	**82.47** ± **0.17**

### 3.4 Ablation study

To study the effect of each component on the results of TriMoGCL, we compare TriMoGCL with 5 variant methods. The results for BC and MC tasks in the MS dataset are shown in Table 3. Class-prototype contrastive loss helps TriMoGCL distinguish motifs with different numbers of edges. Edge pooling enhances the semantic representation of triplets for the triangle which contains a complex and dense topological structure. Node contrastive learning improves the model for recognizing the sparse triplets in dense motifs. As a pivotal feature filter, node pooling maps all triplet features into one embedding space, exploiting the knowledge shared between classes to dramatically improve model performance. GCN enables the model to achieve respectable performance by implementing the common neighbor rule. More experimental results about graph encoder, edge pooling, and contrastive learning are shown in the SD. Overall, TriMoGCL achieves the best performance based on all blocks.

**Table 3.** The ablation results of TriMoGCL for BC (AUC metric) and MC tasks in MS dataset.^a^

	Mi-AUPR	Ma-F1	Triangle	Dise–star	Drug–star	Gene–star	Drug–dise	Gene–dsie	Gene–drug
Ours	92.60±0.14	70.44±0.74	83.43±4.78	91.56±0.88	91.42±1.18	90.38±1.37	98.52±1.62	91.85±0.51	97.87±0.72
V4 = ours w/o PCL	92.43±0.12	69.83±0.85							
V3 = V4 w/o EP	91.12±0.08	67.48±1.06	82.14±4.68	90.98±1.12	91.97±1.25	90.59±1.09	98.66±1.74	91.27±0.55	97.77±0.58
V2 = V3 w/o CL	89.27±0.12	64.10±1.08	80.97±4.68	88.59±1.73	88.22±1.51	88.24±1.11	97.76±1.94	88.33±0.78	96.37±1.05
V1 = V2 w/o NP	84.84±0.12	58.97±0.43	74.85±5.56	88.31±1.88	87.15±1.26	84.94±1.66	97.84±1.94	86.61±0.60	95.87±0.84
V0 = V1 w/o GCN	80.21±0.76	42.44±3.75	67.27±6.89	72.63±2.22	82.40±2.55	78.75±1.82	77.58±3.97	80.60±0.56	93.70±1.00

a PCL: prototype contrastive loss, EP: edge pooling, CL: contrastive learning, NP: node pooling.

### 3.5 Case study

We use TriMoGCL to predict new triangle relationships. The formula of the query is to list which drugs treat disease A by acting on gene B. Two situations are considered: A=Breast Tumor (BT), B=Tumor protein p53 (TP53) and A=Rheumatoid Arthritis (RA), B=Interleukin (IL)-6. Disease A, other neighbor genes, and all drugs are combined into a training dataset, i.e. $t=(BT,\mathrm{Gen}e_{i},\mathrm{Dru}g_{j})$. The label is 1 if *t* is a triangle and otherwise it is 0. We rank predicted probabilities and confirm which triplets in the top 10 form triangles via the Pubmed website. Six triplets in the top 10 are verified that $\mathrm{Dru}g_{j}$ is related to BT by acting on TP53. Seven triplets in the top 10 are verified that $\mathrm{Dru}g_{j}$ is related to RA by acting on IL-6. For instance, it has been verified that Curcumin decreased the expression of TP53 gene in the luminal MCF-7 cell line, and may be of considerable value in synergistic therapy of breast cancer to reduce the associated toxicity with the use of drugs (Quispe-Soto and Calaf 2016). Epigallocatechin-3-gallate (EGCG), an anti-inflammatory compound found in green tea, inhibits IL-1beta-induced IL-6 production and transsignaling in RA synovial fibroblasts, and thus EGCG may be a potential therapeutic agent for RA (Ahmed *et al.* 2008). Detailed results are shown in the SD.

## 4 Discussion

**Figure 3.**
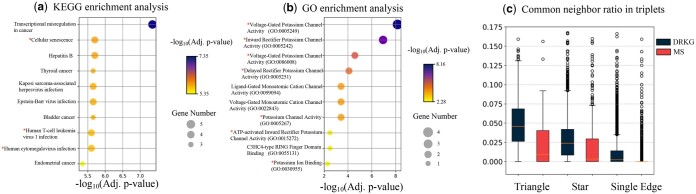
(a) KEGG pathway analysis for Heart Arrest. (b) GO Molecular Function (MF)-enrichment for Potassium. (c) Common neighbor ratios in triplets of each motif.

To analyze the paradigms of motifs, we count the proportion of common neighbors between any two nodes of the triplet. Figure 3 shows that the proportion of common neighbors is higher in triangles than in other motifs, which indicates that having more common neighbors is one of the characteristics of triangles. Aggregating the features of neighbors on the global network makes the common neighbor mechanism work best through GCN.

For instance, in the DRKG dataset, the triangle [Disease: Heart Arrest (HA), Drug: Aspirin, Gene: IL6] has the common neighbor ratio 1.23% (HA-Aspirin), 5.18% (HA-IL6), 7.91% (Aspirin-IL6). The main cause of HA is atherosclerotic heart disease, and low-dose aspirin usage can reduce the risk for HA (Siegel 2015). To demonstrate the importance of neighbors in motif prediction, we conduct gene enrichment for common gene neighbors of HA and aspirin. Among the top 10 enriched KEGG pathways (Xie *et al.* 2021) in common genes and IL6, Cellular senescence, Human T-cell leukemia virus 1 (HTLV-1) infection, and Human cytomegalovirus (CMV) infection are confirmed to be related to HA and aspirin. Senescence of vascular smooth muscle cells contributes to atherosclerosis, and aspirin, as an experimental longevity drug, can suppress cellular senescence to prevent atherosclerosis (Pulakat and Chen 2020, Xiang *et al.* 2022). It’s evidenced that both HTLV-1 and CMV infections activate nuclear factor κB (NF-κB) to cause atherosclerosis (Popović *et al.* 2012, Shimizu *et al.* 2021). Aspirin markedly reduce NF-κB activation and IKKβ kinase activity that is the key protein in regulating IκBα protein levels (Li and Gaynor 1999). The above literature demonstrates that aspirin prevents atherosclerosis and reduces the risk of HA through common gene-enriched pathways i.e. cellular senescence, viral infections.

In addition, TriMoGCL is able to identify the triangle (Disease: Andersen Syndrome, Drug: Potassium, Gene: KCNJ2) with a common neighbor ratio of 0. Andersen Syndrome (AS) is inherited in an autosomal dominant fashion and is caused by mutations in the KCNJ2 gene (Pérez-Riera *et al.* 2021). Drug flecainide is the only neighbor of AS in the network with training triplets. The intersection of Flecainide and Potassium’s gene neighbors includes KCNH2, KCNA5, KCNJ11, and KCNJ2. Mutation in KCNH2 is also related to AS (Polyak *et al.* 2020). GO Molecular Function (MF)-enrichment is performed to examine the neighboring genes. Figure 3 shows the resulting top 10 MFs based on adjusted *P*-values and gene neighbor set captures potassium-related MFs. That is, AS is linked to KCNH2 and Potassium through the neighbors of the Flecainide. In the disease neighbor of KCNH2 and Potassium, the top five diseases that are similar to AS include Long QT Syndrome, Arrhythmias, Cardiac, Heart Diseases, Atrial Fibrillation, and Ventricular Fibrillation. Among them, Atrial Fibrillation and ventricular Fibrillation can be regarded as symptoms of AS. AS is one of Long QT Syndrome. The levels of Arrhythmias, Cardiac, and Heart Diseases are superior to AS. Thus, the disease can be linked to KCNH2 and Potassium through a disease similarity matrix as well.

Furthermore, through studying the feature similarity of triplets, we find that edge pooling exhibits essence for motif prediction in the MS dataset. Supplementary Figure S2 shows that the feature $Z^{B}$ of node pooling exhibits obvious intra-class similarity, except on gene–star and gene–dise motifs. Whereas, the intra-class similarity of feature $Z^{E}$ of edge pooling on both gene–star and gene–dise motifs compensates for the lack of node pooling.

Finally, we discuss the relationship between the triplet motifs and the first-order logic (FOL) queries, and compare the performance of two multi-hop reasoning methods (Ren and Leskovec 2020, Zhang *et al.* 2021) on the BC task (in SD). FOL-based queries cannot fully describe triplet motifs, as the two are different tasks. Therefore, TriMoGCL cannot be replaced by the multi-hop reasoning methods.

## 5 Conclusion

In this paper, we propose a graph contrastive learning-based method (TriMoGCL) to predict seven motif types of (Disease–Drug–Gene) triplets. TriMoGCL applies a graph convolutional encoder on a global network for node learning, which enables the model to achieve respectable performance by implementing the common neighbor rule. Node pooling extracts context information among all triplets as shared knowledge between triplet motifs. Edge pooling exploits transition vectors to construct semantic information different from that of node pooling for enriching triplet features. By reducing redundancy, contrastive learning can refine the context information of triplets of triangle and star motifs, and enhance the ability of representing triplets containing sparse context information. In general, TriMoGCL not only achieves the best performance in two datasets and two tasks, but also reveals new disease–drug–gene triangles for understanding pharmacological mechanisms. Finally, we analyze the paradigms of the triangle motif under the common neighbor rule. In the future, we will mine the new structural properties of triangle motif with large language models in multi-classification task to explain more pharmacological mechanisms.

## Supplementary Material

btaf023_Supplementary_Data

## Data Availability

The codes and data underlying this article are available at https://github.com/zhanglabNKU/TriMoGCL and https://doi.org/10.5281/zenodo.14633572.
