# Supplementary material for: A comprehensive graph neural network method for predicting triplet motifs in disease–drug–gene interactions
Source: Bioinformatics. 2025 Jan 20;41(2):btaf023. doi: 10.1093/bioinformatics/btaf023 (PMC11796092; doi:10.1093/bioinformatics/btaf023)
Supplement: btaf023_Supplementary_Data [file btaf023_supplementary_data.zip › 3343f_supplemental data.pdf]

Supplementary Data

A Comprehensive Graph Neural Network Method for Predicting Triplet Motifs in Disease-Drug-Gene Interactions

Chuanze Kang[1], Zonghuan Liu[1], Han Zhang[1*]
[1] College of Artificial Intelligence, Nankai University, Tianjin, China
[*] zhanghan@nankai.edu.cn

Contents

1.Parameters of baseline methods 3
2.The pseudocode of constructing initial gene features 5
3.Replace GCN with GAT 6
4.Use parameterized modules in edge pooling....7
5.Link prediction methods with probability product to predict triplet motif 8
6.Relationship between the multi-reasoning and the prediction of triplet motif 10
7.Parameter analysis for contrastive learning 13
8.Case study 16

List of Figures

Fig. S 1 The BC results of TriMoGCL and baselines in MS dataset. In each subplot, the left plane is the AUC result and the right plane is the AUPR result. 12
Fig. S 2 The intra-class similarity for triplet feature obtained by node pooling, edge pooling, and final representation. 12
Fig. S 3. The MC results of TriMoGCL under different parameters with τ set {0.1, 1, 10, 1000} and λ set {0.1, 0.5, 1, 2}. 13
Fig. S 4. The BC results of TriMoGCL under different parameters with τ set {0.1, 1, 10, 1000} and λ = 0.1. 14
Fig. S 5. The MC results of prototype contrastive learning and feature masking under different parameters with τ set {0.1, 1, 10, 1000} and λ = 0.1. 15

List of Tables

Table S 1. The experimental results of TriMoGCL with GAT for MC task in MS dataset. 6
Table S 2. The experimental results of TriMoGCL with GAT for MC task in DRKG dataset. 6
Table S 3. The AUC results of TriMoGCL with GAT for BC task in MS dataset. 6
Table S 4. The AUPR results of TriMoGCL with GAT for BC task in MS dataset. 6
Table S 5. The AUC results of TriMoGCL with GAT for BC task in DRKG dataset. 6
Table S 6. The AUPR results of TriMoGCL with GAT for BC task in DRKG dataset. 6
Table S 7. The experimental results of different modules in edge pooling for MC task in MS

dataset. 7
Table S 8. The experimental results of different modules in edge pooling for MC task in MS dataset. 7
Table S 9. The AUC results of different modules in edge pooling for BC task in MS dataset. 7
Table S 10. The AUPR results of different modules in edge pooling for BC task in MS dataset 7
Table S 11. The AUC results of different modules in edge pooling for BC task in DRKG dataset 7
Table S 12. The AUPR results of different modules in edge pooling for BC task in DRKG dataset 7
Table S 13.The experimental results of Link prediction methods with probability product for BC task (triangle) in MS dataset. 8
Table S 14. The experimental results of Link prediction methods with probability product for BC task (triangle) in DRKG dataset..8
Table S 15. The experimental results of Link prediction methods with probability product for MC task in MS dataset. 9
Table S 16. The experimental results of Link prediction methods with probability product for MC task in DRKG dataset..9
Table S 17. The relationship between triplet motif and FOL query. 10
Table S 18. The AUC results of Cone and BetaE for BC task in MS dataset..........................11
Table S 19. The AUPR results of Cone and BetaE for BC task in MS dataset. 11
Table S 20.The AUC results of Cone and BetaE for BC task in DRKG dataset. 11
Table S 21. The AUPR results of Cone and BetaE for BC task in DRKG dataset. 11
Table S 22. The new predicted triangle relationships for {A=Breast Tumor (BT), B=Tumor protein p53 (TP53)}. 16
Table S 23.The new predicted triangle relationships for {A=Rheumatoid Arthritis (RA), B=Interleukin (IL)-6}...16

1.Parameters of baseline methods

Table S1: The parameters of baseline methods.

Methods	Parameters
Random Forest (RF)	The number of trees in the forest is 100. The initial features of the three nodes are concatenated as the input of RF.
MLP	The initial features of the three nodes are concatenated as the input. A three-layer fully-connected network is used to encode triplet features. The input size is 256*3. The hidden dimension of the network is {256*2, 256, 256}.<br>The classifier is the linear layer with output size 7 (or 2).<br>The learning rate is 0.005. The batch size is 5000. The epoch number is 100.
N2V-MLP	Node2vec is used to pre-train node features based on the training graph network.<br>The embedding dimension for node2vec is 256. The walk length is 20.<br>The number of walks to sample for each node is 10.<br>The number of negative samples to use for each positive sample is 1.<br>MLP is used as the classifier to predict the motif of triplet.<br>Pre-training features of three nodes are concatenated as the input.<br>The number of layers in MLP is 2.<br>The hidden dimension of MLP is {256*2, 256,} and the output size is 7 (or 2).<br>For DRKG, the learning rate is 0.0005, the batch size is 2000, and the epoch number is 100.<br>For MS, the learning rate is 0.001, the batch size is 1000, and the epoch number is 50.
TriSAGE	Graph Attention Network is used as the auto-encoder to pre-train the node features.<br>The input is the initial node features. The number of GAT layers is 2. The hidden dimension is {16, 16}. The number of heads is 4. The multi-head attentions are concatenated.<br>The number of decoder layers is 2. The hidden dimension is {64}. The output size is the dimension of the initial features.<br>Pre-training features of three nodes are concatenated as the triplet features.<br>To create a graph with triplets as nodes, the 5 nearest points are selected as neighbors of the node based on the triplet features.<br>SAGE is used to learn the triplet features through graph convolution on the created graph.<br>The number of layers is 2. The hidden dimension is {64, 32}. The output size is 7 (or 2).<br>For DRKG, the learning rate is 0.0005, the batch size is 5000, and the epoch number is 100.<br>For MS, the learning rate is 0.0001, the batch size is 1000, and the epoch number is 100.
TriNet	4 hypergraphs are constructed based on triplets of the triangle, dise-star, drug-star, gene-star motifs.<br>4 hypergraph convolutions are used to learn node features.<br>The hidden dimensions of 4 hypergraph convolutional layers are 256.<br>Then, a self-attention is used to fuse node features from 4 hypergraphs.<br>The number of heads in self-attention is 8. The hidden dimension of self-attention is 8.<br>A linear layer is used as a pooling operator to fuse features of three nodes for classification.<br>For DRKG, the learning rate is 0.0005, the batch size is 5000, and the epoch number is 100.<br>For MS, the learning rate is 0.0001, the batch size is 1000, and the epoch number is 100.
MCHNN	The learning rate is 0.001, the batch size is 10000, the hidden dimension is 256, and the epoch number is 1000.

SEAM	Node2vec:<br>The embedding dimension of node2vec is 32. The walk length is 10.<br>The number of walks to sample for each node is 10.<br>The actual context size which is considered for positive samples is 5.<br>The number of negative samples used for each positive sample is 3.<br>Encoder:<br>The number of hops is 2. The hidden dimension is 64. The number of graph convolutional layers is 2. The k of TopKPooling is 10. The kernel size of 1d convolutional is 4.<br>For DRKG, the learning rate is 0.00005, the batch size is 32, and the epoch number is 32.<br>For MS, the learning rate is 0.00005, the batch size is 32, and the epoch number is 20.

2.The pseudocode of constructing initial gene features

Algorithm 1 Constructing Initial Gene Features

Input: Set of gene nodes $Gene = \{g_1, g_2, \dots, g_n\}$, List of gene features P_{gene}.

Output: Initial gene feature X_{gene}

For $g \in Gene$ **do**

$S_g = Query(Chr, Chr_{start}, Chr_{stop})$ *Query the NCBI database using gene ID g to fetch the corresponding chromatin sequence input.*

$\text{FastaFile} = Chr.fasta$ *Obtain the fasta file of chromatin sequence.*

$Extractor = \text{FastaStringExtractor}(\text{FastaFile})$ *Initialize the sequence extractor.*

$Seq_g = Extractor.extract(Chr_{start}, Chr_{stop})$ *Extract the corresponding sequence of gene* g.

$Tracks_g = Enformer.predict(Seq_g)$ *Use the Enformer model to predict genomic tracks (*$896 \times 5{,}313$ *for human genes and* $896 \times 1{,}643$ *for mouse genes).*

$P_g = PCA.fit(Tracks_g)$ *Apply Principal Component Analysis (PCA) to reduce the number of genomic tracks from 5,313 (or 1,643) to 100.*

Add P_g in list P_{gene}.

Train a convolutional neural network (CNN)-based autoencoder:

The structure and parameters of auto-encoder:

Encoder:

Layer 1: Conv2D(in_dim=1, out_dim=16, kernel_size=4, stride=2, padding=1)

Layer 2: Conv2D(in_dim=16, out_dim=32, kernel_size=4, stride=2, padding=2)

Layer 3: Linear(32*224*25, 8192)

Layer 4: Linear(8192, 1024)

Decoder:

Layer1: Linear(1024, 8192)

Layer2: Linear(8192, 32*224*25)

Layer 3: ConvTranspose2D(in_dim=32, out_dim=16, stride=2, padding=2)

Layer 4: ConvTranspose2D(in_dim=16, out_dim=1, kernel_size=4, stride=2, padding=1)

Input: P_{gene} matrices for all genes.

Output: Encoded gene features X_{gene}.

3.Replace GCN with GAT

We have replaced GCN with GAT and provide the experimental results as follows. GAT doesn't improve the performance of triplet prediction in DRKG dataset. Edge attention can improve the performance of model in small-scale dataset, but suffer from noisy edges in the large-scale dataset.

Table S 1. The experimental results of TriMoGCL with GAT for MC task in MS dataset.

MS	Micro-AUPR	Macro-AUC	Ma-F1	ACC
Ours	92.6±0.14	95.39±0.07	70.44±0.74	85.96±0.25
GAT	93.38±0.44	96.54±0.31	70.77±1.63	86.25±0.64

Table S 2. The experimental results of TriMoGCL with GAT for MC task in DRKG dataset.

DRKG	Micro-AUPR	Macro-AUC	Ma-F1	ACC
Ours	90±0.07	97.3±0.02	78.58±0.18	82.47±0.17
GAT	89.39±0.26	97.14±0.05	77.12±0.48	81.25±0.36

Table S 3. The AUC results of TriMoGCL with GAT for BC task in MS dataset.

MS	triangle	dise-star	drug-star	gene-star	drug-dise	gene-dsie	gene-drug
Ours	83.43±4.78	91.56±0.88	91.42±1.18	90.38±1.37	98.52±1.62	91.85±0.51	97.87±0.72
GAT	83.94±4.33	91.64±0.95	92.75±1.04	91.87±1.28	99.39±0.71	91.67±0.24	98.32±0.52

Table S 4. The AUPR results of TriMoGCL with GAT for BC task in MS dataset.

MS	triangle	dise-star	drug-star	gene-star	drug-dise	gene-dsie	gene-drug
Ours	82.73±5.01	90.73±1.35	90.27±2.45	89.48±1.92	98.51±1.62	90.5±0.76	97.54±0.98
GAT	82.56±2.9	90.83±1.71	92.32±1.23	90.94±2.01	99.44±0.62	90.12±0.58	97.91±0.72

Table S 5. The AUC results of TriMoGCL with GAT for BC task in DRKG dataset.

DRKG	triangle	dise-star	drug-star	gene-star	drug-dise	gene-dsie	gene-drug
Ours	94.15±0.35	95.88±0.29	93.74±0.45	95.37±0.25	98.75±0.31	97.57±0.2	98.61±0.12
GAT	92.08±0.39	95.91±0.47	93.4±0.44	95.56±0.3	99.01±0.32	97.65±0.17	98.41±0.13

Table S 6. The AUPR results of TriMoGCL with GAT for BC task in DRKG dataset.

DRKG	triangle	dise-star	drug-star	gene-star	drug-dise	gene-dsie	gene-drug
Ours	93.95±0.48	95.09±0.53	93.4±0.55	94.97±0.36	98.2±0.63	97.13±0.28	98.38±0.18
GAT	91.39±0.65	94.81±0.91	92.77±0.71	94.7±0.37	98.82±0.41	97.38±0.22	98.17±0.16

4.Use parameterized modules in edge pooling.

We have considered parameterized modules and provided the experimental results as follows. CNN has a negative effect on the binary-class problem. Although RNN has steady performance on binary-class problem, it reduces the performance on the multi-class problem for MS dataset. Both introduce extra parameters but fail to improve the results of the model.

Table S 7. The experimental results of different modules in edge pooling for MC task in MS dataset.

MS	Micro-AUPR	Macro-AUC	Ma-F1	ACC
Ours	92.6±0.14	95.39±0.07	70.44±0.74	85.96±0.25
CNN	91.92±0.66	95.57±0.43	68.35±2.26	85.67±1.02
RNN	91.42±0.85	95.15±0.43	66.56±2.49	85.41±0.61

Table S 8. The experimental results of different modules in edge pooling for MC task in MS dataset.

DRKG	Micro-AUPR	Macro-AUC	Ma-F1	ACC
Ours	90.00±0.07	97.30±0.02	78.58±0.18	82.47±0.17
CNN	89.52±0.29	97.16±0.09	77.89±0.43	82.08±0.26
RNN	89.38±0.26	97.11±0.09	77.68±0.53	81.87±0.31

Table S 9. The AUC results of different modules in edge pooling for BC task in MS dataset.

MS	triangle	dise-star	drug-star	gene-star	drug-dise	gene-dsie	gene-drug
Ours	83.43±4.78	91.56±0.88	91.42±1.18	90.38±1.37	98.52±1.62	91.85±0.51	97.87±0.72
CNN	81.16±5.59	88.86±2.02	87.69±1.43	88.31±1.52	98.23±2.12	84.48±0.59	96.69±0.82
RNN	83.09±5.32	91.45±1.27	92.27±0.78	90.23±1.29	98.51±1.48	91.23±0.57	97.94±0.61

Table S 10. The AUPR results of different modules in edge pooling for BC task in MS dataset

MS	triangle	dise-star	drug-star	gene-star	drug-dise	gene-dsie	gene-drug
Ours	82.73±5.01	90.73±1.35	90.27±2.45	89.48±1.92	98.51±1.62	90.5±0.76	97.54±0.98
CNN	81.79±4.87	88.27±2.53	84.86±1.84	87.83±1.42	97.51±4.14	83.54±0.69	96.34±1.08
RNN	81.19±6.22	90.74±1.83	91.23±1.59	89.58±1.93	98.36±1.77	89.81±0.88	97.61±0.88

Table S 11. The AUC results of different modules in edge pooling for BC task in DRKG dataset

DRKG	triangle	dise-star	drug-star	gene-star	drug-dise	gene-dsie	gene-drug
Ours	94.15±0.35	95.88±0.29	93.74±0.45	95.37±0.25	98.75±0.31	97.57±0.2	98.61±0.12
CNN	90.82±0.46	94.87±0.46	92.08±0.65	93.88±0.4	98.5±0.31	95.79±0.26	97.57±0.19
RNN	94.1±0.24	96.07±0.36	93.87±0.42	95.48±0.28	98.83±0.3	97.58±0.18	98.57±0.13

Table S 12. The AUPR results of different modules in edge pooling for BC task in DRKG dataset

DRKG	triangle	dise-star	drug-star	gene-star	drug-dise	gene-dsie	gene-drug
Ours	93.95±0.48	95.09±0.53	93.4±0.55	94.97±0.36	98.2±0.63	97.13±0.28	98.38±0.18
CNN	89.91±0.76	93.86±0.79	91.32±0.62	93.05±0.58	98.06±0.34	94.87±0.32	97.02±0.37
RNN	93.87±0.31	95.33±0.55	93.55±0.53	95.3±0.29	98.35±0.58	97.13±0.28	98.35±0.19

5.Link prediction methods with probability product to predict triplet motif

To highlight the necessity of the TriMoGCL, we propose incorporating a comparison with two probability combination baselines where the likelihood of a triplet motif is derived by multiplying the probabilities of its constituent edges. For a fair comparison, we apply state-of-the-art link prediction (LP) methods to independently predict drug-disease, gene-disease, and drug-gene relationships. The likelihood of triplet motif will then be derived from the product of these probabilities.

Two baselines are from the leaderboards for link property prediction on the Open Graph Benchmark (OGB) website: OGB-GCN (NIPS 2020 [1]) and Neural Common Neighbor (NCN, ICLR 2024 [2]). Contrastive learning is used to improve the quality of the node representation of OGB-GCN for a fair comparison as well. For the binary-classification task, the AUC and AUPR results of the triangle are shown as follows. For the multi-classification task, we select the mi-AUPR, ma-AUC, ma-F1, and ACC as metrics and show the overall results across motifs. In followed tables, 'independently' represents that LP methods predict three relationships in three independent training phases, and 'dependently' represents that LP methods predict three relationships in one training phase.

Table S 13.The experimental results of Link prediction methods with probability product for BC task (triangle) in MS dataset.

MS	AUC	AUPR
Ours	83.43±4.78	82.73±5.01
OGB-GCN- independently	65.44±7.83	64.14±7.23
OGB-GCN- dependently	63.90±9.42	63.80±8.75
NCN- independently	63.22±5.66	66.86±3.34
NCN- dependently	61.14±5.29	63.40±2.87

Table S 14. The experimental results of Link prediction methods with probability product for BC task (triangle) in DRKG dataset.

DRKG	AUC	AUPR
Ours	94.15±0.35	93.95±0.48
OGB-GCN- independently	86.10±1.06	87.26±1.05
OGB-GCN- dependently	86.38±1.06	87.52±1.11
NCN- independently	86.99±0.96	87.08±0.83
NCN- dependently	87.09±0.54	86.97±0.54

Table S 15. The experimental results of Link prediction methods with probability product for MC task in MS dataset.

MS	Micro-AUPR	Macro-AUC	Ma-F1	ACC
Ours	92.60±0.14	95.39±0.07	70.44±0.74	85.96±0.25
OGB-GCN- independently	65.67±1.00	79.36±1.21	44.57±1.45	67.14±0.97
OGB-GCN- dependently	68.93±1.28	80.28±0.96	44.67±1.04	68.59±0.75
NCN- independently	57.94±2.31	77.76±1.38	43.05±1.37	63.63±1.73
NCN- dependently	51.14±6.01	74.17±3.40	38.14±2.69	53.03±7.83

Table S 16. The experimental results of Link prediction methods with probability product for MC task in DRKG dataset.

DRKG	Micro-AUPR	Macro-AUC	Ma-F1	ACC
Ours	90.00±0.07	97.30±0.02	78.58±0.18	82.47±0.17
OGB-GCN- independently	68.09±0.23	86.38±0.21	63.77±0.22	69.43±0.21
OGB-GCN- dependently	67.34±0.84	86.23±0.30	62.49±0.60	68.66±0.62
NCN- independently	63.45±1.02	86.63±0.45	61.64±0.76	66.10±0.62
NCN- dependently	68.69±4.01	90.37±1.24	60.23±1.42	67.14±1.77

The methods of probability combination cannot predict complex motifs due to their inability to capture higher-order dependencies and motif-level context. However, TriMoGCL is designed to excel in these scenarios, which demonstrates its superiority and necessity for comprehensive prediction of triplet motifs. Triplets represent the intricate relationships between entities that cannot be inferred by independently combining pairwise link predictions. TriMoGCL leverages both information from global and local contexts to derive discriminative features specific to the motif structures.

In general, the existing link prediction methods cannot predict triplet motifs in disease-drug-gene interactions. Thus, it is necessary to establish a deep learning paradigm specific to the prediction of triplet motifs and propose new methods to learn the mapping between features and motifs of triplets.

[1] Hu, W., et al., 2020. Open graph benchmark: Datasets for machine learning on graphs. Advances in neural information processing systems, 33, pp.22118-22133.
[2] Wang X.Y., et al., 2024. Neural Common Neighbor with Completion for Link Prediction. In The Twelfth International Conference on Learning Representations.

6.Relationship between the multi-reasoning and the prediction of triplet motif

In this section, we discuss the relationship between triplet motif and FOL-based query[1].

Definition 1 (First-order logic query). A first-order logic query q consists of a non-variable anchor entity set $V_a \subset V$, existentially quantified bound variables $V_1, \dots, V_k$ and a single target variable$V_?$ which provides the query answer. The disjunctive normal form of a logical query q is a disjunction of one or more conjunctions.

$$q[V_?] = V_?. \exists V_1, \dots, V_k: c_1 \vee c_2 \vee \dots \vee c_n$$

1. Each c represents a conjunctive query with one or more literals e. $c_i = e_{i1} \wedge e_{i2} \wedge \dots \wedge e_{in}$.
2. Each literal e represents an atomic formula or its negation. $e_{ij} = r(v_a, V) or \neg r(v_a, V) or\ r(V', V) or \neg r(V', V)$, where $v_a \in V_a$, $V \in \{V_?, V_1, \dots, V_k\}$, $V' \in \{V_1, \dots, V_k\}$, $V \neq V'$, $r \in R$. Each relation type $r \in R$ is a binary function $r: V \times V = \{True, False\}$ that indicates (directed) edges of relation type r between pairs of entities.

Based on the definition of FOL, the FOL query is to find the answer set that satisfies the multi-hop reasoning. The FOL query consists of the non-variable anchor entity, existentially quantified bound variables, and a single target variable. Limited to the definition, the FOL formula for the triangular triplet is “List the disease treated by drug A and regulated by genes that are targeted by drug A.” FOL doesn’t incorporate the conditions among bound variables in the query of the formula, so it cannot fully describe the triangular triplet prediction.

To evaluate the performance of multi-hop reasoning methods on the triplet motif prediction, we adjust the FOL formula to the binary-class prediction of the triplet motif.

Table S 17. The relationship between triplet motif and FOL query.

Motif	FOL query
Triangle	List the disease treated by drug A and regulated by genes that are targeted by drug A.
Dise-Star	List the gene regulating disease A and not targeted by drugs that treat disease A.
Drug-Star	List the disease treated by drug A and not regulated by genes that targeted drug A.
Gene-Star	List the disease regulated by gene A and not treated by drugs that target gene A.
Drug-Dise	List the gene not regulating disease A and not targeted by drugs that treats disease A.
Gene-Dise	List the drug not treating disease A and not targeting genes that regulates disease A.
Drug-Gene	List the disease not treated by drug A and not regulated by genes that are targeted by drug A.

We compare two state-of-the-art FOL-based reasoning models BetaE[1] and ConE[2]. The results are shown as follows. BetaE and ConE suffer from noisy graphs and complex motifs with similar semantics. In contrast, TriMoGCL, with its graph convolutional encoder and contrastive learning framework, excels at overcoming these problems. The performance of FOL-based reasoning models BetaE and ConE may be improved by adding conditions among bound variables in the query of the formula in the future.

Table S 18. The AUC results of Cone and BetaE for BC task in MS dataset.

MS	triangle	dise-star	drug-star	gene-star	drug-dise	gene-dsie	gene-drug
Ours	83.43±4.78	91.56±0.88	91.42±1.18	90.38±1.37	98.52±1.62	91.85±0.51	97.87±0.72
Cone	68.99±1.15	89.13±0.59	85.98±0.68	71.17±0.63	95.16±0.95	81.20±0.29	94.08±0.48
BeatE	69.36±3.64	85.95±0.59	86.13±0.58	67.68±1.19	91.99±1.59	79.73±0.09	93.81±0.51

Table S 19. The AUPR results of Cone and BetaE for BC task in MS dataset.

MS	triangle	dise-star	drug-star	gene-star	drug-dise	gene-dsie	gene-drug
Ours	82.73±5.01	90.73±1.35	90.27±2.45	89.48±1.92	98.51±1.62	90.50±0.76	97.54±0.98
Cone	71.88±0.95	89.47±0.65	86.99±0.78	69.95±0.99	95.82±0.88	78.94±0.44	93.30±0.52
BeatE	71.86±4.48	85.57±0.44	86.08±0.91	63.77±1.07	91.16±2.87	76.22±0.16	93.76±0.51

Table S 20.The AUC results of Cone and BetaE for BC task in DRKG dataset.

DRKG	triangle	dise-star	drug-star	gene-star	drug-dise	gene-dsie	gene-drug
Ours	94.15±0.35	95.88±0.29	93.74±0.45	95.37±0.25	98.75±0.31	97.57±0.20	98.61±0.12
Cone	84.85±0.15	92.94±0.17	83.29±0.19	84.76±0.13	96.09±0.19	91.57±0.07	95.36±0.07
BeatE	83.29±0.53	92.34±0.96	83.29±0.25	81.52±0.48	93.34±0.87	91.54±0.08	95.05±0.3

Table S 21. The AUPR results of Cone and BetaE for BC task in DRKG dataset.

DRKG	triangle	dise-star	drug-star	gene-star	drug-dise	gene-dsie	gene-drug
Ours	93.95±0.48	95.09±0.53	93.40±0.55	94.97±0.36	98.20±0.63	97.13±0.28	98.38±0.18
Cone	83.51±0.40	91.18±0.24	80.85±0.38	81.94±0.32	94.37±0.62	90.29±0.11	94.35±0.13
BeatE	81.84±0.53	89.72±0.96	80.54±0.25	78.43±0.48	89.8±0.87	89.5±0.08	93.86±0.30

[1] Ren, H., & Leskovec, J. (2020). Beta embeddings for multi-hop logical reasoning in knowledge graphs. Advances in Neural Information Processing Systems, 33, 19716-19726.

[2] Zhang, Z., Wang, J., Chen, J., Ji, S., & Wu, F. (2021). Cone: Cone embeddings for multi-hop reasoning over knowledge graphs. Advances in Neural Information Processing Systems, 34, 19172-19183.

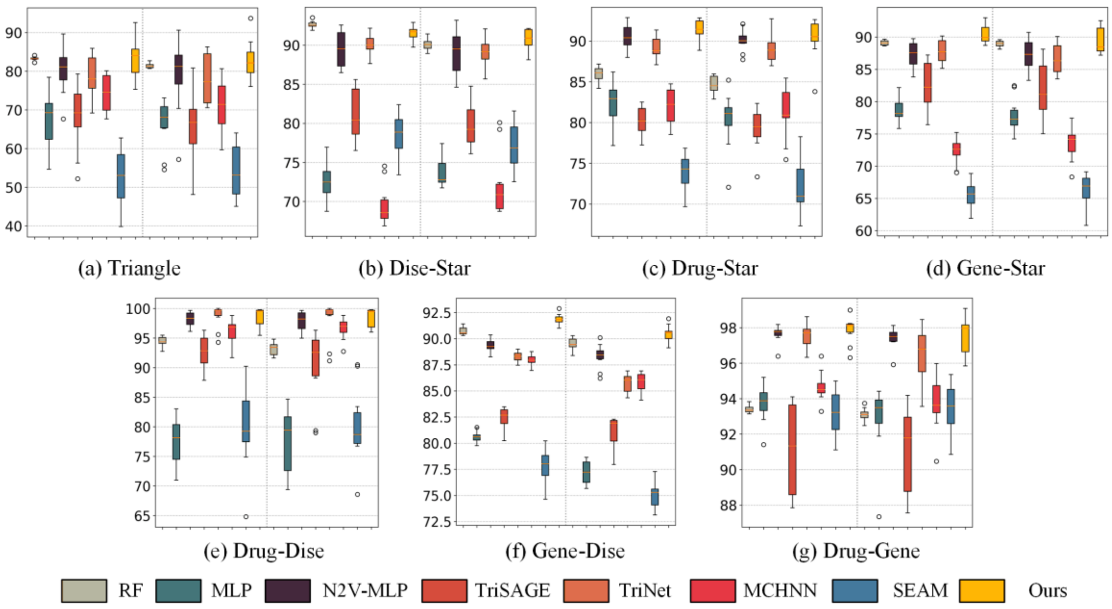

Fig. S 1 The BC results of TriMoGCL and baselines in MS dataset. In each subplot, the left plane is the AUC result and the right plane is the AUPR result.

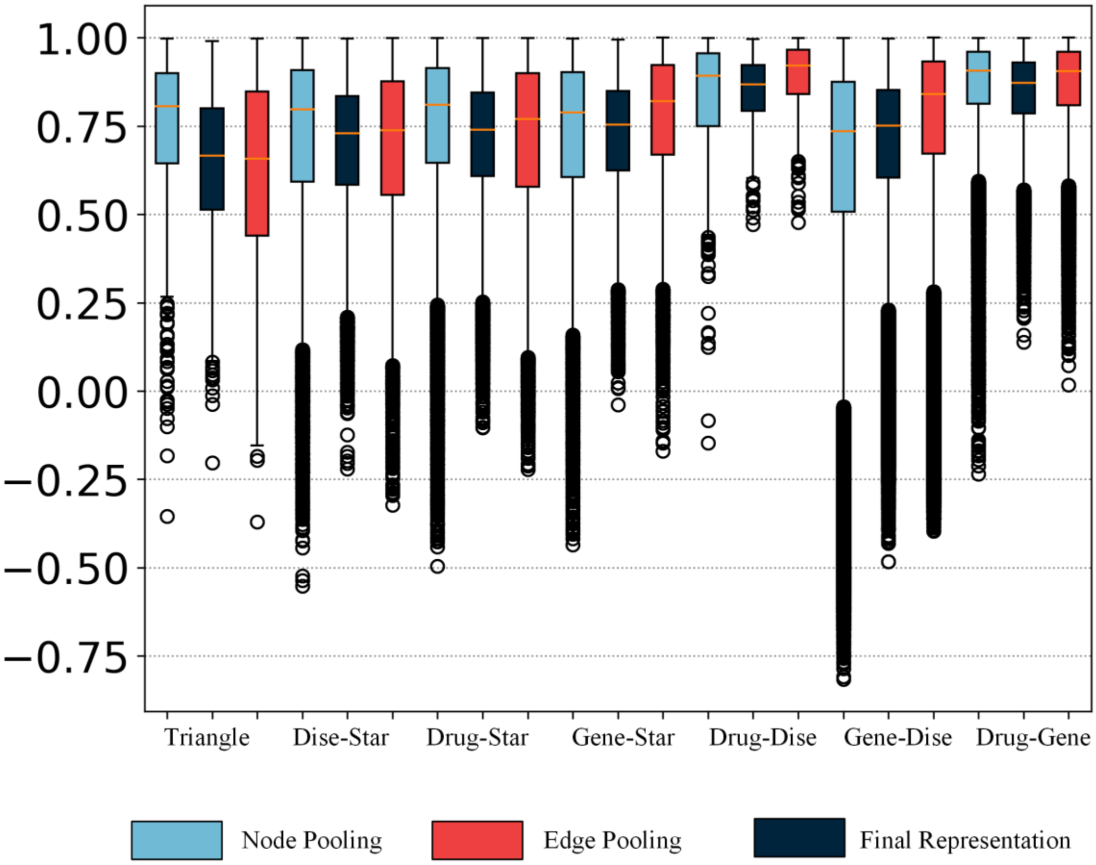

Fig. S 2 The intra-class similarity for triplet feature obtained by node pooling, edge pooling, and final representation.

7.Parameter analysis for contrastive learning

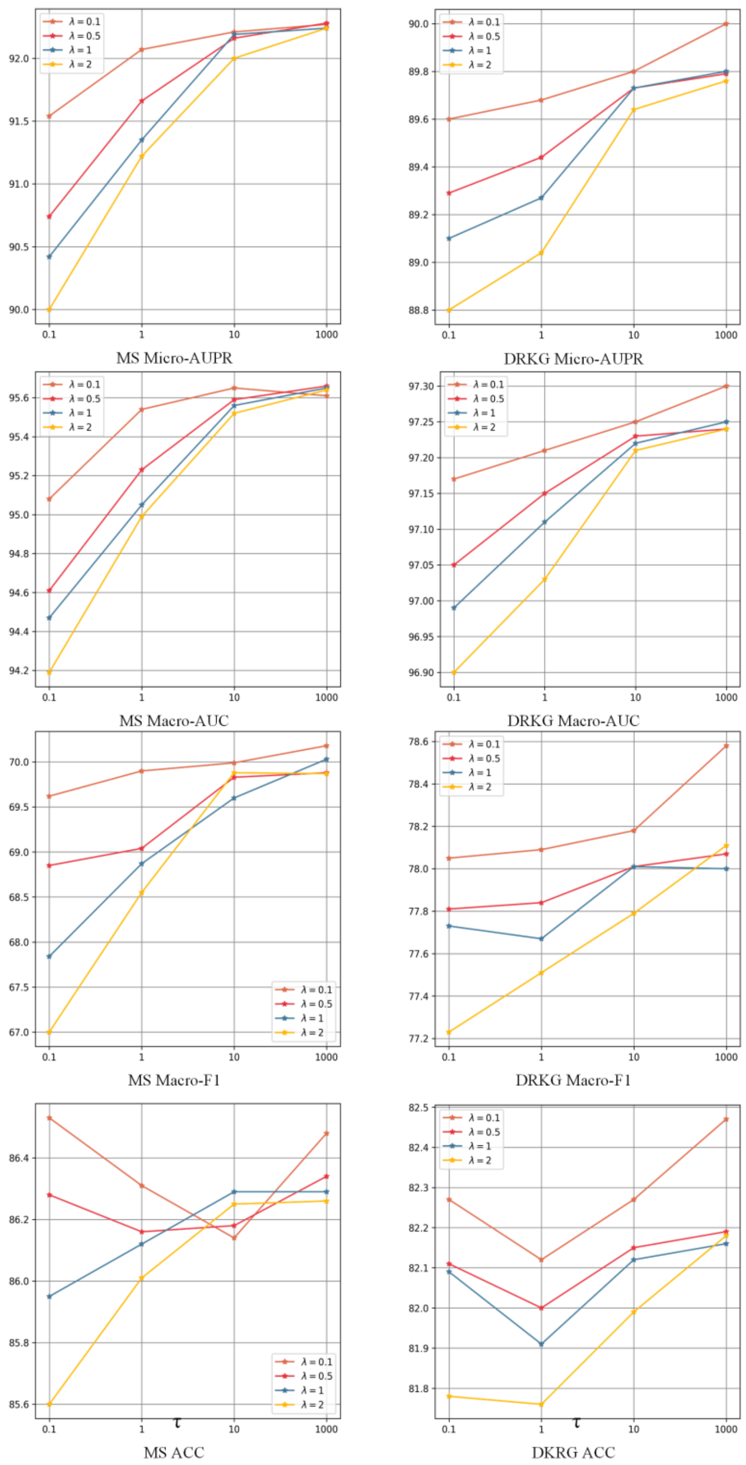

Fig. S 3. The MC results of TriMoGCL under different parameters with τ set {0.1, 1, 10, 1000} and λ set {0.1, 0.5, 1, 2}.

For MC task, the increase of τ improves the performance of the model, but the increase of λ severely reduces the performance of model. Therefore, we fix $\lambda = 0.1$ in subsequent experiments.

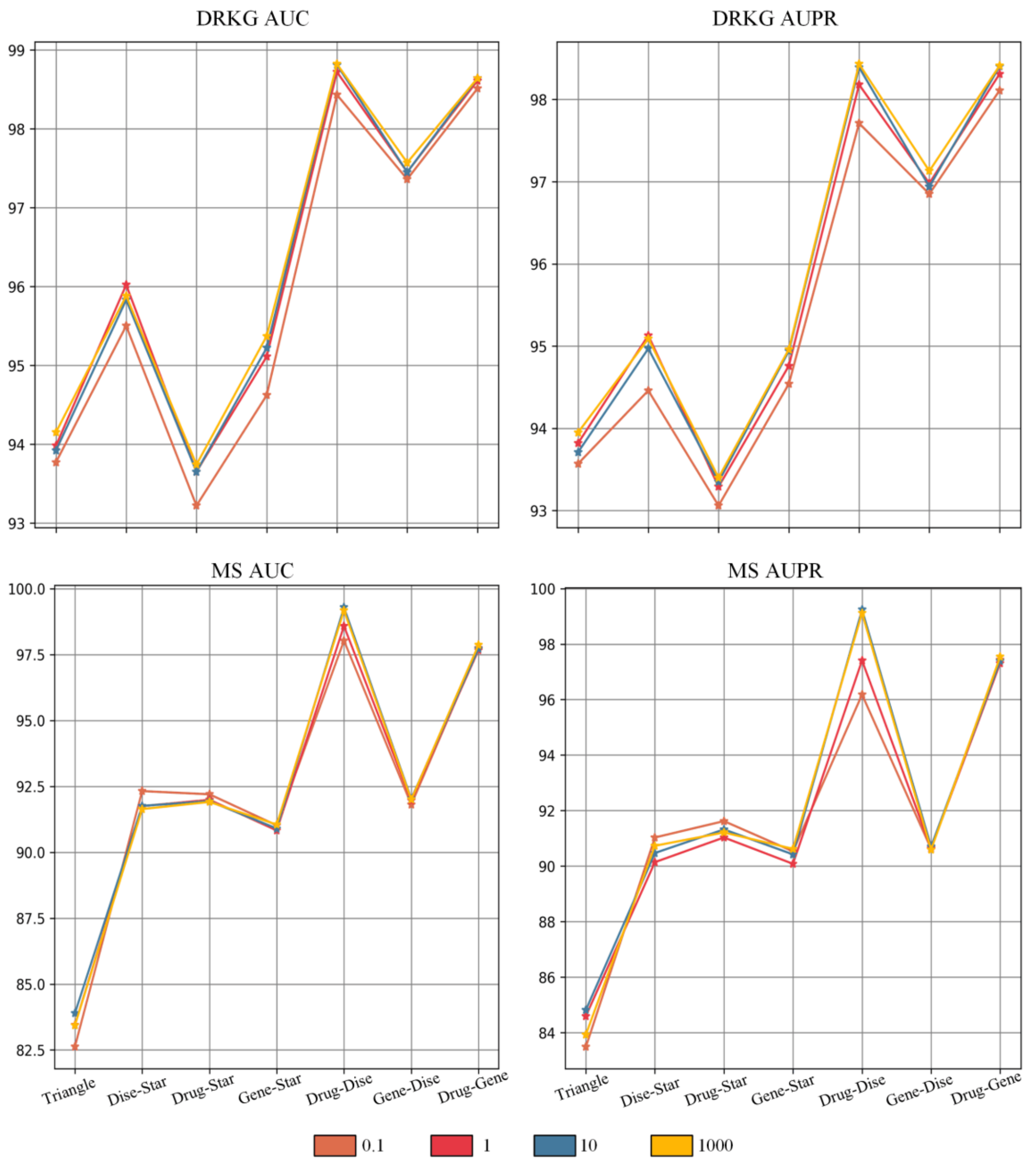

Fig. S 4. The BC results of TriMoGCL under different parameters with τ set {0.1, 1, 10, 1000} and $\lambda =$ 0.1.

For BC task, changes in τ do not affect the performance of the model.

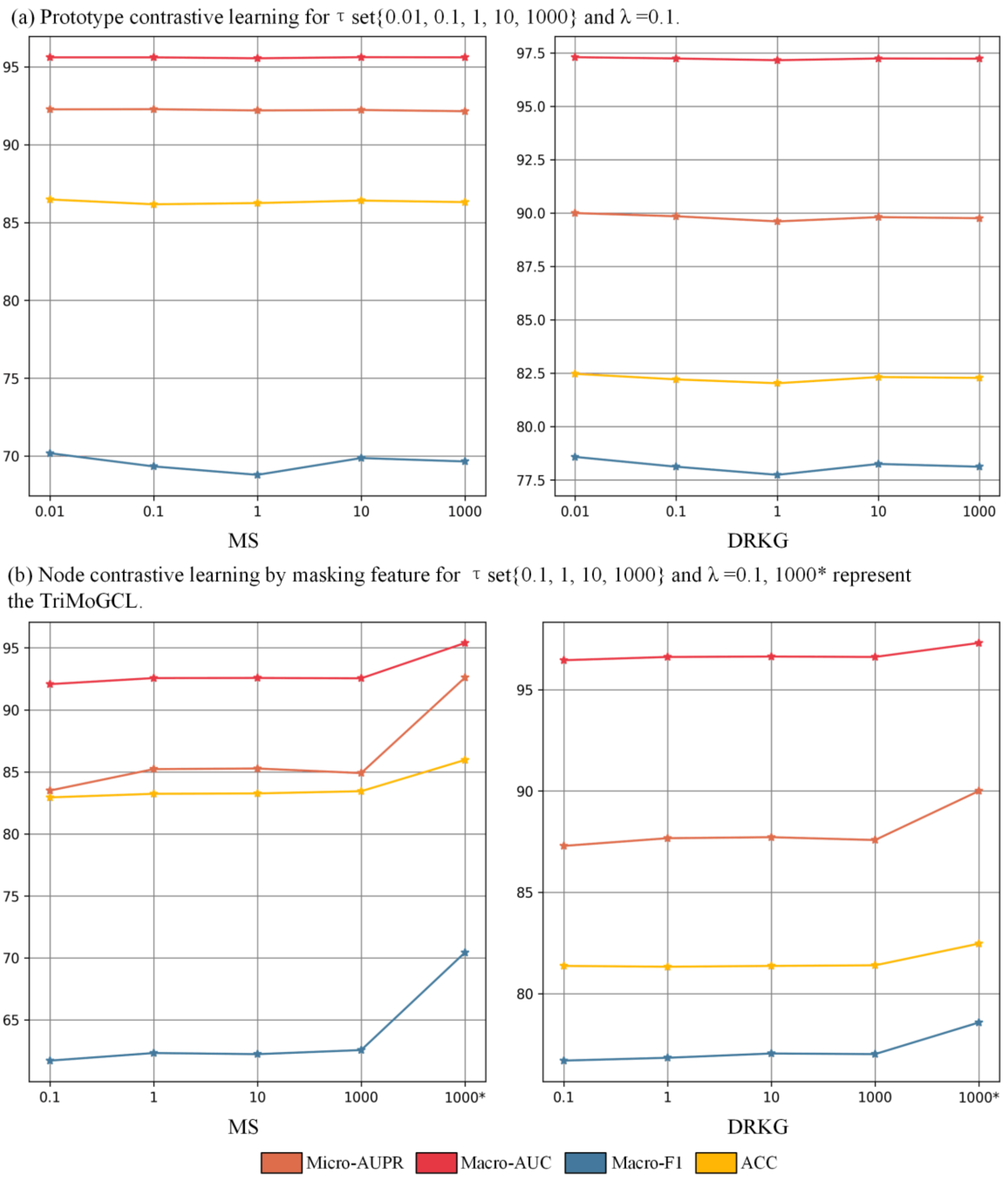

Fig. S 5. The MC results of prototype contrastive learning and feature masking under different parameters with τ set {0.1, 1, 10, 1000} and $\lambda = 0.1$.

For the MC task, changes in τ of the prototype contrastive learning do not affect the performance of the model.

Feature masking is another common approach to generating new views for contrastive learning. Results show that feature masking suffers from severe degradation in performance. However, the key factors hindering prediction are the redundant context information and motif imbalance problem caused by dense edges. The feature masking experiment not only validates the good quality of initial features but also shows that the noise of dense edges leads to the failure of imputation for features by neighborhood aggregation.

8.Case study

We use TriMoGCL to predict new triangular relationships. The formula of the query is that list drugs treat disease A by acting on gene B. Two situations are considered: {A=Breast Tumor (BT), B=Tumor protein p53 (TP53)} and {A=Rheumatoid Arthritis (RA), B=Interleukin (IL)-6}.

Disease A, other neighbor genes, and all drugs are combined into a training dataset, i.e. $t = (BT, Gene_i, Drug_j)$. The label is 1 if t is a triangle and 0 otherwise. We rank predicted probabilities and confirm which triplets in the top 10 form triangles via the Pubmed website.

6 triplets in the top 10 are verified that $\mathrm{Drug_j}$ is related to BT by acting on TP53. 7 triplets in the top 10 are verified that $\mathrm{Drug_j}$ is related to RA by acting on IL-6. Detailed results are shown in Tables S22 and S23.

Table S 22. The new predicted triangle relationships for {A=Breast Tumor (BT), B=Tumor protein p53 (TP53)}.

Rank	Disease	Gene	Drug	Pubmed
1	Breast-Tumor	TP53	Cholesterol	26350565
2	Breast-Tumor	TP53	Curcumin	27779649
3	Breast-Tumor	TP53	Progesterone	37044296
4	Breast-Tumor	TP53	Fluorouracil	14654539
6	Breast-Tumor	TP53	Hydrocortisone	30325501
7	Breast-Tumor	TP53	Genistein	30737644

Table S 23.The new predicted triangle relationships for {A=Rheumatoid Arthritis (RA), B=Interleukin (IL)-6}.

Rank	Disease	Gene	Drug	Pubmed
1	Arthritis, Rheumatoid	IL6	Methylprednisolone	31897527
3	Arthritis, Rheumatoid	IL6	Vitamin C	38599880
4	Arthritis, Rheumatoid	IL6	Betamethasone	23365744
6	Arthritis, Rheumatoid	IL6	Vitamin D3	32056400
8	Arthritis, Rheumatoid	IL6	Histamine	28446753
9	Arthritis, Rheumatoid	IL6	Epigallocatechin-3-gallate	18796608
10	Arthritis, Rheumatoid	IL6	Pentoxifylline	27671331

For instance, it has been verified that curcumin combined with paclitaxel decreased p53 gene expression in the luminal MCF-7 cell line. Curcumin alone and combined with paclitaxel increased p53 gene expression in MDA-MB-231. However, either substance alone and combined increased Bax protein expression corroborating the apoptotic effect of these substances. It can be concluded that curcumin may be of considerable value in synergistic therapy of breast cancer to reduce the associated toxicity with the use of drugs [1].

Genistein (GEN) and cisplatin (CIS) showed synergistic effects only in ERβ-deficient cells. This effect mainly cause G2 phase to be arresting and resulted in apoptosis induction with the upregulation of P21 and Bax/Bcl-2 protein levels. Besides, P53 expression was strikingly suppressed in ERβ-deficient cells. This indicated ERβ pathway deficiency might enhance GEN-

CIS bioactivity via the downregulation of P53. In summary, our data imply that daily intake of GEN-rich diet could collaborate with CIS anti-tumor treatment in ERα-/ERβ- breast cancer cases [2].

Epigallocatechin-3-gallate (EGCG), an anti-inflammatory compound found in green tea, inhibits IL-1beta-induced IL-6 production and transsignaling in RA synovial fibroblasts by inducing alternative splicing of gp130 mRNA, resulting in enhanced sgp130 production. Results from in vivo studies using a rat adjuvant-induced arthritis model showed specific inhibition of IL-6 levels in the serum and joints of EGCG-treated rats by 28% and 40%, respectively, with concomitant amelioration of rat adjuvant-induced arthritis. The results of these studies provide previously undescribed evidence of IL-6 synthesis and transsignaling inhibition by EGCG with a unique mechanism of sgp130 up-regulation, and thus hold promise as a potential therapeutic agent for RA [3].

Treatment of collagen-induced arthritis mice with vitamin C effectively rescued the gut microbiota imbalance and suppressed the inflammatory response associated with RA, and effectively alleviated arthritis symptoms in mice in which levels of the pro-inflammatory cytokines IL-6 and TNF-α were specifically reduced. In conclusion, our results demonstrate the potential of vitamin C as a potential therapeutic choice for RA [4].

[1] Quispe-Soto ET, Calaf GM. Effect of curcumin and paclitaxel on breast carcinogenesis. Int J Oncol. 2016 Dec;49(6):2569-2577. doi: 10.3892/ijo.2016.3741. Epub 2016 Oct 19. PMID: 27779649.
[2] Liu R, Xu X, Liang C, Chen X, Yu X, Zhong H, Xu W, Cheng Y, Wang W, Wu Y, Yu L, Hu X. ERβ modulates genistein's cisplatin-enhancing activities in breast cancer MDA-MB-231 cells via P53-independent pathway. Mol Cell Biochem. 2019 Jun;456(1-2):205-216. doi: 10.1007/s11010-019-03505-y. Epub 2019 Feb 8. PMID: 30737644.
[3] Ahmed S, Marotte H, Kwan K, Ruth JH, Campbell PL, Rabquer BJ, Pakozdi A, Koch AE. Epigallocatechin-3-gallate inhibits IL-6 synthesis and suppresses transsignaling by enhancing soluble gp130 production. Proc Natl Acad Sci U S A. 2008 Sep 23;105(38):14692-7. doi: 10.1073/pnas.0802675105. Epub 2008 Sep 16. PMID: 18796608; PMCID: PMC2567200.
[4] Zhang Y, Zhen S, Xu H, Sun S, Wang Z, Li M, Zou L, Zhang Y, Zhao Y, Cui Y, Han J. Vitamin C alleviates rheumatoid arthritis by modulating gut microbiota balance. Biosci Trends. 2024 Jun 6;18(2):187-194. doi: 10.5582/bst.2024.01037. Epub 2024 Apr 10. PMID: 38599880.
